# Policy challenges for cancer research: a call to arms

**DOI:** 10.3332/ecancer.2008.53

**Published:** 2007-09-18

**Authors:** R Sullivan

**Affiliations:** Chairman, European Cancer Research Managers Forum and Director, Clinical Centres Cancer Research UK

## Abstract

Research has delivered remarkable benefits for cancer patients and their families since James Watson and Francis Crick wrote the now immortal line, **‘We wish to propose a structure for the salt of deoxyribonucleic acid’** thus setting the molecular foundations for the modern era of cancer control. The pace of technological innovation from fundamental scientific discoveries to the policy impact of huge population studies has been breathtaking. One has only to contrast a paper on the treatment of solid epithelial cancers written by Henri Tagnon and colleagues in 1966 (*Eur J Cancer* **2** 51–7) with the myriad of chemotherapeutic approaches at the oncologists disposal today. Inevitably, as the tide of research has risen so it has bought the flotsam and jetsam of regulations and policies. Some have been helpful, many pointless and too many actually harmful. Naturally, some of these regulatory and general policies (by this I mean those concerned with funding, structure and organization) have been specifically targeted at cancer research, e.g. US National Cancer Act 1971, whilst others have been a product of the general regulatory environment with indirect consequences for cancer research, e.g. EU Data Protection Directive 1995. Policy issues thus cover a vast terrain criss-crossed by complex interdependencies between scientific areas, countries S&T policies and socio-political constructs. Unfortunately, there has been little attention paid to the consequences of these policy issues from which the research community has, by and large, been passenger rather than driver.

Global investment in cancer research is now at unprecedented levels. The recently published report by the European Cancer Research Managers Forum has found some 14 billion euros being annually spent worldwide on cancer research (this figure includes industry but overall probably underestimates spend by at least one billion [**2**]). With the ageing demographics of developed countries and the catch-up effect in developing countries, the rising burden of cancer is driving research activity in cancer ever upwards. Opportunities for delivering even greater measures for preventing and controlling cancer abound, but the shackles of bureaucracy (stifling regulations and poor research policies) threaten this future more than ever—**‘Man is born free and everywhere he is in chains’**. Jean-Jacques Rousseau’s quote could equally be applied to spirit of research creativity in today’s environment. So what are the main issues and what is to be done?

## Tower of Babel

**Figure 1:**
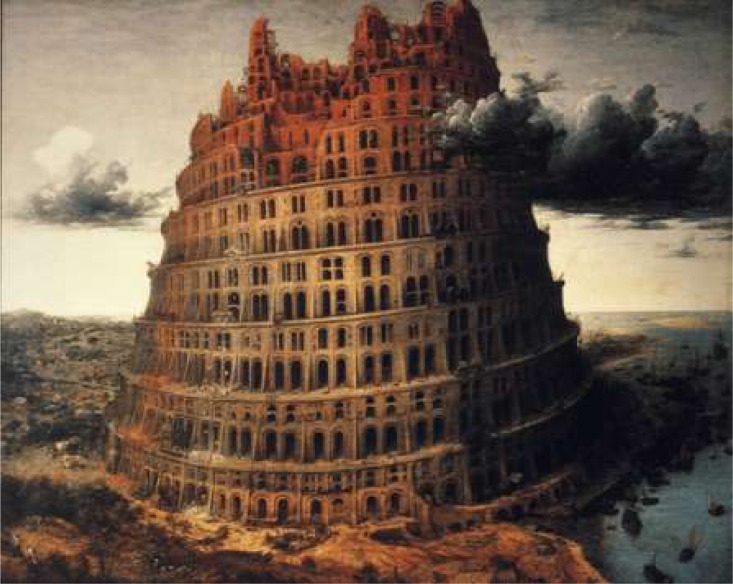
The Tower of Babel by Pieter Brueghel the Elder (1563).

The story of the Tower of Babel (probably derived from the ancient Sumerian myths around their great towers, or ziggurats) is well known (Figure 1). Divine intervention shattered humanities single language into a multiplicity of tongues, thus sowing confusion (balal is the Hebrew word ‘to confuse’). To describe the structural and advocacy constructs in Europe and the United States as ‘Towers of Babel’ underestimate the scale of the problem. There is little interest in cancer-research policymaking circles about questions of dependence and interdependence, asymmetrical relations amongst funders and countries, centre-periphery relations or indeed in research imperialism. And yet considering the scale of cancer research activity, investment and the socio-political prominence that it enjoys such questions must be addressed. Without this understanding it is seriously questionable whether any major funder, country or representational body can honestly construct a strategy that has external validity.

Funding for cancer research remains buoyant in overall terms but serious questions remain about the structures through which this funding flows. At the European level, research policy has been expressed since 1983 through five-year ‘framework programmes’. Disease-specific research has had to stand in line with the broader European goals. Whether this is actually the right thing to do as a matter of principle is a matter of fierce debate [3] but what is clear is that, as with all politically centred strategies, long-term planning and commitment has not been Europe’s forte, to the detriment of cancer research whose programmes of research and deliverables stretch well beyond political life cycles. The failure to commit to Europe against cancer and the network of cancer registries are just two such examples.

The creation of the European Research Area and the ‘3% objective’ heralded change with increased emphasis on really adding European value and networking existing Member State Research Programmes [4]. In the end, however, the bottom line prevailed and instead of supporting the broad church of research, it has instead overwhelmingly concentrated on areas with perceived high industrial value. Perhaps no surprise given the overt political objectives of the Union, but this misses the bulk of cancer research that has high societal value. It remains to be seen whether the Seventh Framework Programme will be more holistic. The confusion of policy messages emanating from Brussels suggests the battle lines are still being drawn although the latest perspective on the European Research Area suggests a move to long-term planning and funding [5]. There is little doubt that after the fiasco of the Article 169 joint-funding initiative (European and Developing Countries Clinical Trials Partnership) [6], the Commission and Council remain wary of complex, disease-hypothecated programmes. Despite this, funds have been delivered for the Innovative Medicines Initiative, although this is again directed at a narrow area of cancer research (new chemical entities, biomarkers, etc) and focused on commercial utility. Applications going forward to the first round of FP7 funding from cancer registries and paediatric oncology (Innovative Therapies for Children with Cancer—ITCC Consortium) will be the real acid test of a policy shift. There is little doubt that Europe is committed to adding value and networking, at least in principle, but the practicalities of putting this into effect within the Byzantine forum of European policymaking are somewhat daunting. Europe is not alone in this respect. In the United States, the Bayh-Dole Act 1980 provided the ultimate experiment in how public institutions could manage public assets as private goods. Almost everyone is now in agreement that the Act, whilst certainly being fiscally beneficial, caused the public benefit of sharing and accessing Intellectual Property to be overridden by private interests to the detriment of research [7]. Examples of this type of misalignment abound in Europe and the United States, products as they are of confused policymaking between separate Directorates or Divisions prosecuting their own agendas with little regard to any coherent integrated strategy. Whilst attempts have been made since the mid-nineties to bring together major cancer policy groups in the United States, for example through the National Cancer Policy Board (then Forum from 2005) [8], Europe remains a mass of Member State and representational groups.

The structural and organizational issues around cancer research policy stretch deeper into individual Member States and representational groups. Following Charles de Gaulle’s maxim that ‘politics is too serious a matter to be left to politicians’, Europe has a vast array of policy active bodies agitating along a broad, but often conflicting and overlapping, front for research and cancer control programmes [9]. There are European bodies that claim to speak for patients, domains of research (e.g. imaging) and professional specialities within cancer. If you then add in the multiple voices at Member State level, particularly the charities, then you truly have a Tower of Babel. No wonder then, with so many voices claiming the high moral ground, confusion reigns even in the most sympathetic high political circles. To a certain extent, competition is good even in policymaking and the advocacy arena; there is much ground to cover and no one has the monopoly on the ultimate vision to defeat cancer. However, if such diversification continues and more political schisms open up between representational bodies, the impact and influence of this community on the socio-political process for allocating funds will be seriously diminished over the next decade. Cancer is not the only healthcare issue; research and progress around correcting the 10/90 gap is rapidly becoming the dominant health and disease priority [10]. Member States have also, by and large, not grasped the need to adequately fund cancer research in their country and/or create a coordinated environment that can at least bring the multiple funders of cancer research into that Member State (exceptions to this are France through L’INCA and the UK’s NCRI). The ECRM survey has found that throughout Europe the individual policies of Member States have led to very low funding allocations for cancer research in many countries. This skews mobility (many countries lose their most valuable investigators), and encourages subsidy-seeking behaviour of the research community in Member States either from industry (thereby only prosecuting a narrow research area) and/or EU funding (thereby reducing the value-adding aspect of this funding source). Confused S&T polices at Member State level, which de-prioritize cancer research, need to be addressed urgently if a broader European vision is to be realized.

### Networks, centres, institutes: organizational policies for cancer research

The organizational structures within which cancer research is conducted are products of the prevailing socio-political policies and healthcare/university systems. How a research community is framed can have dramatic effects on the productivity, focus and overall impact. Because of the breadth of cancer research nearly all organizational models been applied in some fashion over the last 50 years. Indeed views on the best way to organize cancer research are as varied as the stars in the sky! One of the most interesting observations as one listens to debates in this policy area is how much, as John Stuart Mills would put it, ‘the othersideness’ of human nature comes through. Rational, evidence-based discussion and constructs are the exception. Perhaps this is to be expected when one considers this is a fundamental social construct; however, much more high-quality intelligence needs to percolate into this arena.

European structures have been dominated either by the historical nature of individual Member States approach to cancer research funding or have fallen in line with the political aims of the European Union. European cancer research structures have been dominated by the emphasis in the framework programmes on networks of excellence and integrated projects. Lately, this has focused almost wholly on public–private partnerships (e.g. IMI). ‘The European Paradox’ [11] (which refers to the fact that Europe plays a leading world role in terms of scientific excellence, but largely fails to convert science-based findings and inventions into wealth-generating innovations) remains a potent driver to these structural polices at European level, despite the fact that this approach essentially cuts out a great deal of research that, whilst of great utility to society, has little commercial value. This policy of networking has not been the rampant success that was originally envisaged. Indeed, the research communities soon found ways of creating superficial networks to access framework funding when, in reality, there was little depth to the research relationships. On the other hand, domains of research that really were networked and/or absolutely required trans-national networking in order to conduct the research in the first place (e.g. large-scale clinical trials [12], particularly in orphan areas and population studies, including infrastructure support) went largely ignored. However, good programmes were funded and the Commission retort to this could easily point the finger at the Council of Ministers and European Parliament for cutting the framework budgets so heavily as to strangle certain promising structural initiatives at birth.

One structural area that has gathered momentum in Europe focuses on the concept of coordination (essentially this is a more proactive form of programmatic networking). In 2001 two initiatives to create a ‘virtual European Cancer Institute’ were started—one under the auspices of a group led by Prof. Thiery Philip (France, CR Leon-Berard) [13], and the other by Profs. Fritz Schroder and Bob Pinedo (a joint FECS-ECRI Programme). Both camps gathered pace and by the end of 2001 both camps were lobbying heavily to have their respective visions funded, at least in a pilot phase under framework 6. These activities led in the following February (2002) to a Commission-sponsored conference, Towards Greater Coherence in Cancer Research, out of which the ECRM was started. It soon became abundantly clear that all players had overestimated the funding that might be available and underestimated the political inertia that had to be overcome to gain any sort of momentum behind such an ambitious plan. Thus by the end of February, there were the first indications that the time for such ambition was probably not right. From 2002 to 2005, the ECRM continued its modest activities and various ERA-NET (European Research Area Network) grants were awarded in cancer, e.g. CoCANCPG (clinical practice guidelines), although the one ERA-NET that could have improved coordination—a proposal to provide an accurate and comprehensive coding and database of all Member State cancer research—was turned down on the grounds of not being exciting enough!

**Figure 2:**
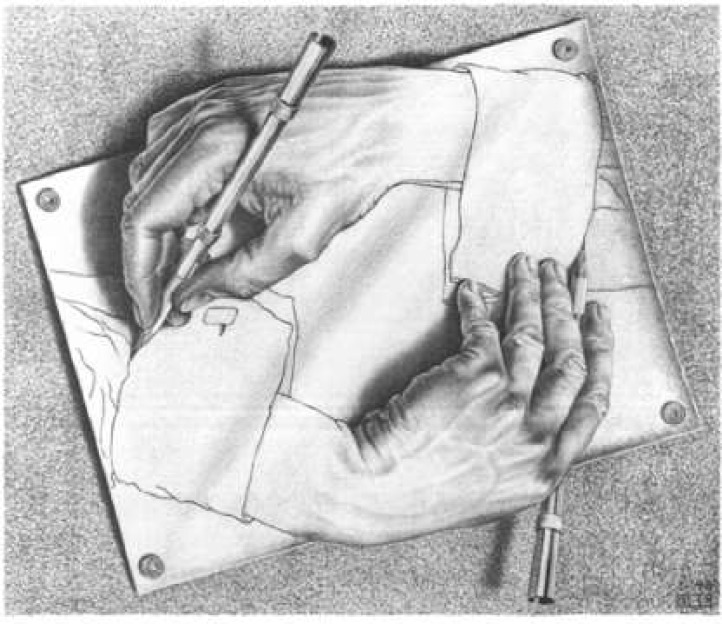
Maurits Cornelis Escher, Drawing Hands (1948).

In 2005, a proposal led by Prof. Peter Boyle to review areas for greater coordination (EUROCAN +) was funded. This initiative will report soon and is likely to conclude that the case for a European Cancer Institute (virtual or otherwise) is still strong. In parallel, a variety of different trans European groups are prosecuting their own strategies—from the EORTC’s Network of Core Institutions to the OECI’s open letter to the Commission offering to further develop the accreditation for European Comprehensive Cancer Centres. Whilst the hybrid approach (centres and networks) is probably the way to go, the multiplicity of participating individuals and groups in these processes poses exactly the same issues faced by the early pioneers for better cancer research organization across Europe. Furthermore, there are some fundamental organizational defects at Member State level that could mean that any ECI is built on foundations of sand. Furthermore, Europe simply does not yet have sufficient understanding of the different models of cancer centres, which have been built up in each Member State; indeed, data that the ECRM will publish next year on EU and US centres indicate a huge heterogeneity in terms of size, productivity, research strengths and cultural paradigms. Unlike the US model where a top-down strategy has been in process since 1971 thus creating a structurally homogenous (but still complex) organization, that of Centres Europe has grown organically from the bottom up which makes any top-down changes hugely difficult to implement. The question also needs to be asked as to whether such structural radicalism is actually needed in Europe. There is a strong case that the US one-structure-fits-all approach has actually reduced overall creativity. Europe may be messy but in terms of cancer research it still works. Fundamentally, the principle players have yet to articulate what the benefits of such an approach will be and how such benefits will actually be made tangible. All this leaves one feeling slightly dizzy as though following the hands drawn by Maurits Escher (Figure 2). In policy terms what is needed is a fixed point of reference to which one can anchor descriptions of cancer research structures to affirm and defend their validity. The structural and organizational needs of cancer research will be many and diverse. As Prof. Tanja Cufer has eloquently pointed out ‘new’ Europe has managed to pioneer some highly forward-thinking structures that provide front-line clinical research even in a country as small as Slovenia (where the population is two million strong) [14]. In the rush for new policies to set the next generation of cancer research centres and their coordination Europe needs to be far more cooperative from a sociological perspective, and rigorous in its analysis of current structures and behaviours. This perspective cannot, must not, be taken for granted. As Humberto Maturana and Francisco Varela have observed, ‘Everything we do is a structural dance in the choreography of coexistence’. [15]

### Regulating cancer research: How far before we say stop?

If this all wasn’t enough to worry about yet again Europe seems to be lurching towards another biomedical regulatory disaster with the proposed EU Physical Agents (EMF) Directive 2004/40/EC that is seeking to limit exposure to a hypothetical threat of electromagnetic radiation. If implemented in its current form it would be a disaster for MRI research and the treatment of cancer patients. There are hopeful signs that the directive will be changed [16] but yet again, and despite the fiasco around the creation of the ‘Clinical Trials’ Directive, the apparatus of European regulatory policymaking has been found wanting.

These latest tribulations can be added to a long list of regulatory policies, mostly directives that have caused nothing but trouble for cancer research (and indeed for all other types of research)—Data Protection, Clinical Trials [17], etc. Because these regulatory policies tend to be anthropocentric, i.e. they deal with matters relating to people, their tissues and medical data—the domains of cancer research that have been hit hardest—they are already those which are the most difficult to conduct, whilst being the most valuable in terms of direct patient benefit. Thus, we have the perverse situation where the emphasis is now on translational and clinical research, whilst at the same stroke the regulatory burden is exponentially increased. The United States and individual Member States fair little better. The former has seen a dramatic increase in regulations, e.g. HIPPA, whilst in the latter case unilateral action, for example in England and Wales against human tissues, has led to its own set of unique problems. At the heart of this lies a fundamental flaw in the way that risk is viewed by policymakers (particularly those in the EU). The concept of the universal precautionary principle, a product of early German and Swedish thinking about environmental policy (and, in light of Rachel Carson’s Silent Springs, this certainly was the correct approach for the environment, that isn’t under contention), became the core principle for managing biomedical risk. However, biomedical research is a very different paradigm from environmental issues and, as such, a precautionary approach leads to ‘precaution without principle’ and a reduction/stoppage of public-benefit research as costs increase (note: despite industry also suffering they are far more capable of passing on the costs to consumers). [18]

Although the cancer research community has finally started to engage on these issues, the business of regulation appears to be an unstoppable juggernaut. Simply put, the over-regulation of science is one of the single biggest threats to cancer research. There are no glib answers to this most dangerous of situations. Globalization of research means that if one continent or country does not do it then another will (embryonic stem cell research moving to Europe is a good example of this effect). In this situation, realism pervades (real-politic) and the situation is a never-ending game of control and escape. Such a situation though is untenable as the cost per unit research continually escalates without any improvement in productivity (this is 101 economics!). Somehow, the prevailing regulatory paradigms must be deconstructed and challenged at every opportunity. Here, the research community must engage the patient advocacy groups to explain why this is not special pleading and drive home the reality of this threat.

## Final thoughts

The reader will not be in the least bit surprised to learn that the policy issues discussed in this short article represent a fraction of the ongoing fun and games. For example, we have not even touched upon the festival of delights that is the regulatory environment around drugs and devices, genetics, stem cells, animal research, healthcare policy and its impact on research, etc. In this sense, cancer research policy can seem distant and daunting; all the more reason not to engage. However, it is the moral responsibility of the research community to engage and even more so to take control. Allowing the media and illiterate political policymakers to dictate the destiny of cancer research fails patients and their families. Never has there been a more urgent need to create a third culture [19] of direct interaction between the research community and public, as well as direct action at the policy level.
